# Evaluation of bacterial diversity of traditional cheese in Tarbagatay Prefecture, China, and its correlation with cheese quality

**DOI:** 10.1002/fsn3.2275

**Published:** 2021-04-09

**Authors:** Kaili Zhang, Mengzhen Jia, Zhuang Guo, Yuhui Li, Baokun Li, Xu Li

**Affiliations:** ^1^ School of Food Science and Technology/Key laboratory of Xinjiang Phytomedicine Resource and Utilization of Ministry of Education Shihezi University Shihezi China; ^2^ Hubei Provincial Engineering and Technology Research Center for Food Ingredients Hu Bei University of Arts and Science Xiangyang China

**Keywords:** bacterial diversity, flavor, Illumina MiSeq high‐throughput sequencing, *Lactobacillus*, *Lactococcus*, traditional handmade cheese

## Abstract

In Xinjiang, China, traditional handmade cheese is made from fresh milk under natural environmental conditions and is a common fermented dairy product in the region. Due to differences in production methods between regions, the research conducted on the bacterial diversity of traditional handmade cheese is not comprehensive. Hence, little is known about the relationship between bacteria and cheese quality. Therefore, in this study, cheese samples from Tarbagatay Prefecture, Xinjiang, were chosen for investigation. The bacteria in 17 cheese samples were analyzed by sequencing 16S rRNA using Illumina MiSeq technology. The results showed that there were two dominant bacterial phyla and six dominant bacterial genera in the cheeses. Of these, *Lactobacillus* and *Lactococcus* displayed the most significant positive correlation with cheese quality. This study provides data to support the improvement of traditional cheese quality via microbial diversity and lays a foundation for the industrialization of traditional cheese production.

## Graphical abstract

Traditional cheese of Xinjiang, China, has a long history and the consumption demand is also increasing. In recent years, people pay more and more attention to the quality and microbial diversity of Xinjiang traditional cheese, which also contains a variety of microbial resources. The results showed that the bacteria in Xinjiang traditional cheese were rich and diverse, and the core dominant genera were Lactobacillus, Streptococcus, Lactococcus, Staphylococcus, Enterococcus, and Acetobacters. This study provides a basis for the industrialization of traditional fermented dairy products.

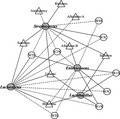

## 1 INTRODUCTION

Cheese is the main product of the developed dairy industry and has high nutritional and economic value. However, due to differences in geographic location and taste preferences, the production process and flavor of cheese vary, and cheese has developed local characteristics (Subramanian et al., 2009; González‐Córdova et al., 2016; Zheng, Liu, Li, et al., 2018). The Kazakhs in Xinjiang, China, have practiced and depended on animal husbandry for generations. Traditional cheese production and consumption has been practiced for thousands of years; the earliest cheese samples in the world have been unearthed in Xinjiang (“Ancient Cheese,” 2014). Kazakh cheese in Tarbagatay, Xinjiang, is also known as Kurut (Tulaxi et al., 2011). Fresh milk and microorganisms from the natural environment are used as a starter for Kazakh cheese. The cheese is handmade and it matures naturally. Because of its unique production process and good taste, Kazakh cheese has always been loved by ethnic minorities in Xinjiang.

As Xinjiang's traditional handmade cheese is mostly made in an open environment, a variety of microorganisms from the environment contribute to the fermentation and post‐ripening of the cheese. Therefore, Xinjiang's traditional cheese is made using a multi‐bacterial fermentation system (Zheng, Liu, Li, et al., 2018; Zheng, Liu, Ren, et al., 2018). The interactions between the various bacteria provide the cheese with a unique flavor and distinctive regional characteristics (Jasmin et al., 2011). Cheese develops its unique flavor and taste during maturation. The dominant microbial strains participate in the fermentation process of cheese, fermenting lactose into lactic acid (Singh et al., 2010), decomposing protein and fat into small molecular flavor compounds, and promoting the maturation of cheese and the formation of flavor substances (Gerrit et al., 2010). The interactions between microorganisms also affect the formation of cheese flavor substances (Ayad et al., 2010). Aldrete et al. (2014) recently used pyrosequencing to analyze 30 cheese samples collected from Mexico, and the results showed that *Streptococcus* and *Lactobacillus* were the dominant genera in the cheese samples. The complex microbiome of a cheese promotes its quality (Schornsteiner et al., 2014). Previous studies (Chmitz‐Esser et al., 2018) have used quantitative PCR technology to analyze the three genera, *Advenella*, *Psychrobacter*, and *Psychroflexus*, to identify genes contributing to cheese flavor. Studies have been performed in an attempt to reveal that there is correlation between the microbial flora and the characteristic flavor of cheese (Aldrete et al., 2014). Therefore, it is particularly important to explore the bacterial diversity in traditional cheeses from Xinjiang. Such investigations will lay a foundation for choosing a good starter for traditional fermented dairy products and will provide insight into the correlation between microbial diversity and traditional cheese flavor.

For traditional fermented foods, traditional pure culture techniques, such as enrichment culture and separation and purification, are generally used to explore the microbial diversity. Such time‐consuming and complicated operations limit the identification of dominant microorganisms in the sample (Mo & Sun, 2012). Therefore, Illumina MiSeq, which is a second‐generation high‐throughput sequencing technique and is accurate, fast, and highly sensitive, has previously been chosen for studying microbial diversity (Bella et al., 2013).

Researchers have used Illumina MiSeq to characterize the microbial diversity in 60 cheese samples (including 12 varieties), and the results showed that the main bacteria present included *Corynebacteriumgroup casei*, *Leucobacter aridicolis*, and *Arthrobacter arilaitensis* (Dugat‐Bony et al., 2016). In another study, high‐throughput sequencing was used to characterize 137 cheeses from 10 countries (Wolfe et al., 2014) and 14 bacterial genera were found. Among them, *Yaniella* and *Nocardiopsis* were found in food microbial ecosystem for the first time, indicating that high‐throughput sequencing has great advantages in detecting the microbial diversity and functional potential of samples. This technology has been widely used to study the microbial community structure of many traditional fermented foods such as tempeh (Chen et al., 2019), sweet rice wine (Cai et al., 2018), and pickles (Lee et al., 2019). The development of these studies provides a firm basis for the analysis of microbial diversity in cheese samples.

In the present study, Illumina MiSeq high‐throughput sequencing technology was used to analyze the bacterial community of traditional cheese from Tarbagatay, Xinjiang. The aim was to explore the correlation between the bacterial community and the taste and flavor of the cheese. In this way, the study will provide a foundation for improving the microbial community structure of traditional fermented dairy products from Xinjiang and provide a basis for screening starters to improve the quality of traditional fermented foods.

## 2 MATERIALS AND METHODS

### 2.1 Cheese samples

The samples were collected from Tarbagatay Prefecture, Xinjiang, China (44.43°N, 84.68°E), which is located in the northwestern region of Xinjiang, adjacent to Yili and Altay. Grasslands account for 46.8% of the entire area, and the luxuriant pastures are good for animal husbandry. Therefore, 17 post‐ripening Kazakh cheeses from different farmers were collected in Tarbagatay Prefecture, and the samples were numbered TC1–TC17 in the order of collection. For each cheese, there were three replicate samples.

### 2.2 Evaluation of cheese taste quality using an electronic tongue

An SA‐402B electronic tongue (Japan Insent Company) equipped with two reference electrodes and five taste sensors was used for taste analysis. During the test, the detection method used by Zhu et al. (2020) was referred to in order to obtain the original data required for the analysis.

### 2.3 Evaluation of cheese flavor quality using an electronic nose

First, 10 g of cheese was placed into a 50 ml sample bottle. Referring to the previously described electronic nose detection method (Zhu et al., 2020), an electronic nose (Airsense, Germany) was used to determine the flavor. The response values were selected at 49 s, 50 s, and 51 s, the average value was calculated, and the operation was repeated three times.

### 2.4 DNA extraction, PCR amplification, and product purification

In this study, a QIAGEN DNeasy mericon Food Kit was used to extract DNA, and the DNA samples were stored at −20°C in preparation for analysis. The DNA extracts were used as the templates, and the universal primers 338F (5'‐ACTCCTACGGGAGGCAGCAG −3') and 806R (5'‐GGACTACHVGGGTWTCTAAT −3') were used for PCR amplification of the 16S rRNA gene of lactic acid bacteria. The amplification conditions were the same as those described by previous researchers (Nacef et al., 2016). The PCR amplification products were sent, using a dry ice‐cold chain, to Shanghai Meiji Biomedical Technology Co., Ltd. where MiSeq high‐throughput sequencing was performed.

### 2.5 Sequence quality control and bioinformatics analysis

The paired‐end sequence data obtained by MiSeq sequencing were spliced and filtered, and the chimera were removed (Haas et al., 2011) for optimization (Kalscheur et al., 2012) and quality control. The QIIME analysis platform (Version 1.7.0) was used to analyze the obtained optimized sequences for species and diversity information. PyNAST was used to align the sequences (Caporaso et al., 2010). UCLUST merging was performed under 100% sequence similarity using the V3–V4 region of the 16S rRNA sequences (Edgar, 2010). Operational taxonomic units (OTUs) were established and ChimeraSlayer (Haas et al., 2011) was used to remove the OTU sequences containing chimeric sequences. Greengenes (DeSantis et al., 2007) and the Ribosomal Database Project (RDP; Cole et al., 2007) were used to conduct homology comparisons to determine the taxonomic classification status of the bacterial OTUs.

Based on the analysis of the phylogenetic tree generated by FastTree software (Price et al., 2009), α‐diversity indicators such as the Chao1 and Shannon indexes were used to evaluate the bacterial diversity and community structure of the traditional Xinjiang cheese samples. Differences in the community structure at different taxonomic levels were also analyzed to evaluate the composition and diversity of the bacterial communities (Wu et al., 2019) in the different cheese samples. These analyses were performed to provide a basis for the subsequent analysis of the relationship between bacterial diversity and cheese quality.

### 2.6 Data analysis

Pearson correlation analysis was used to analyze the electronic tongue and electronic nose data from the cheese samples. The analyzed data were used to draw a principal component analysis chart using Origin 2017C software (Origin Lab). Origin 2017C software (Origin Lab) was also used to draw graphs and histograms from the high‐throughput sequencing results. SAS was used for correlation analysis, and Cytoscape and R language were used for mapping. All the high‐throughput sequence data in this study have been submitted to MG‐RAST database (http://www.mg‐rast.org), ID number is mgp97991.

## 3 RESULTS AND DISCUSSION

### 3.1 Determination of the taste of traditional Xinjiang cheese

**FIGURE 1:**
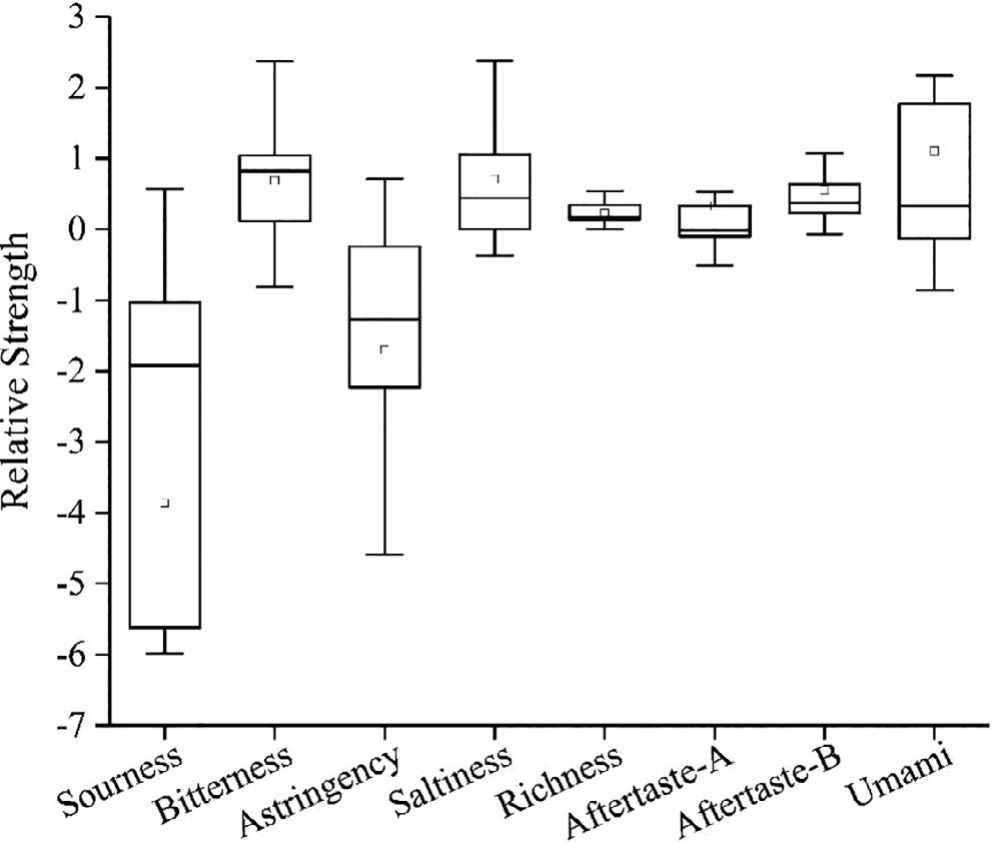
Box diagram of relative strength values of cheese flavor indexes (*n* = 17)

In this study, 17 cheese samples were evaluated for taste quality. The relative intensity values of each cheese taste index are shown in Figure 1. Among the eight taste quality indicators used for the cheese in the electronic tongue test, the relative intensity difference of seven indicators was >1. Only one indicator displayed a difference <1. This indicated that most of the flavors in the cheese could be distinguished according to sensory scores (Kobayashi et al., 2010). The relative intensity differences of the sourness and astringency indexes were the largest at 6.55 and 5.31, respectively. Among them, the sour taste of cheese in this study is similar to the results of some researchers on cheese electronic tongues (Cong et al., 2015). These were followed by bitterness, umami, saltness, aftertaste‐B, and aftertaste‐A, with relative intensity differences at 3.18, 3.02, 2.75, 1.14, and 1.04, respectively. Only the relative intensity difference of richness was less than 1.0, at 0.54. It can be seen that richness cannot be identified by sensory indicators and will not affect cheese preference. Overall, it was found that there were some differences in the taste quality of the 17 cheese samples.

### 3.2 Determination of aromatic substances in traditional Xinjiang cheese

An electronic nose with ten sensors was used to evaluate cheese flavor (Table 1). Among the sensors, W5S, W1S, W1W, and W2S displayed high response values to cheese flavor; their average response values were between 8.43 and 29.5. In contrast, the average response values of sensors W1C, W3C, W5C, W2W, W6S, and W3S were low, at between 0.27 and 1.84. The larger the response value, the greater the concentration of the volatile flavor substances corresponding to the sensor in the cheese (Rudnitskaya & Legin, 2008). Therefore, compared with ethanol, traditional Xinjiang cheese has fewer aromatic substances. This low content of aromatic substances is related to the production technology and the demand of local minorities for specific cheese tastes (Zheng, Liu, Ren, et al., 2018). These results will further support the standardization and industrialization of traditional fermented food. Thus, overall there were differences in both the tastes and aromas of the different cheeses. Studies have shown that these differences are related to the main bacteria present in the cheese (Gerrit et al., 2010; Londoño‐Zapata et al., 2017).

**TABLE 1:** Difference analysis of electronic nose flavor indicators in cheese samples (*n* = 17)

Sensor name	General description	Average (median, minimum–maximum)
W1C	Aromatic compounds	0.27 (0.27, 0.14–0.35)
W5S	Very sensitive; broad‐range sensitivity; reacts to nitrogen oxides; very sensitive with negative signal	18.07 (16.78, 7.89–53.96)
W3C	Ammonia; used as a sensor for aromatic compounds	0.34 (0.34, 0.2–0.43)
W6S	Mainly hydrogen, selectively (breath gases)	1.18 (1.17, 1.11–1.38)
W5C	Alkenes; aromatic compounds; fewer polar compounds	0.32 (0.33, 0.17–0.43)
W1S	Sensitive to methane; broad range	25.67 (24.02, 14.35–55.25)
W1W	Reacts to sulfur compounds; sensitive to many terpenes and sulfur organic compounds that are important for smell; limonene; pyridine	29.5 (26.88, 16.77–85.17)
W2S	Detects alcohols; partially aromatic compounds; broad range	8.43 (7.67, 4.76–19.59)
W2W	Aromatic compounds; sulfur organic compounds	1.84 (1.74, 1.58–3.1)
W3S	Reacts to high concentrations; sometimes very selective (methane)	1.37 (1.32, 1.24–1.92)

### 3.3 Sequence analysis and diversity statistics based on MiSeq high‐throughput sequencing technology

Using the QIIME platform, bioinformatics analysis was further conducted on the high‐quality sequences. After using PyNAST to align the sequences, a UCLUST division was performed according to 100% and 97% similarities. A total of 16,639 OTUs were obtained, with an average of 961 OTUs per sample.

The sequence and classification information for each cheese sample are shown in Table 2. It was found that the number of bacterial sequences detected in the different cheese samples varied. The number of bacteria within each microbial classification level also varied among the samples. Sample TC10 displayed the largest number of sequences, and sample TC11 displayed the largest number of OTUs. Sample TC9 displayed more microorganisms at the phylum, class, order, family, and genus levels than the other samples. Using alpha diversity analysis, it was found that when the number of sequences was 38,010, TC6 had the highest Chao 1 index and TC11 had the highest Shannon index among the 17 cheeses. It could be seen that TC6 was associated with the greatest richness of bacteria, and TC11 was associated with the greatest bacterial diversity. In contrast, TC16 displayed the lowest Chao 1 and Shannon indexes. It can thus be concluded that the bacterial abundance and diversity was the lowest in TC16.

**TABLE 2:** Sequence information and taxonomic status statistics of the 17 samples of traditional Xinjiang cheese (TC1–17)

Sample	Number of reads	Number of OTUs	Phylum	Class	Order	Family	Genus	Chao1 Index^a^	Shannon Index^a^
TC1	46,523	1,100	3	4	7	9	10	1675	3.56
TC2	44,462	647	2	3	6	10	14	964	1.91
TC3	42,015	984	2	3	8	11	20	1645	3.20
TC4	47,677	1,174	2	3	5	9	14	1826	3.30
TC5	45,125	1,120	4	6	11	14	21	1788	3.17
TC6	50,849	1,249	4	5	11	13	21	1896	3.67
TC7	50,533	1,143	3	4	8	11	14	1798	3.84
TC8	51,758	876	3	5	6	9	11	1518	2.66
TC9	45,689	647	5	12	19	23	32	1,259	2.39
TC10	58,670	933	3	3	4	7	8	1505	2.65
TC11	53,552	1,382	3	6	11	17	24	1817	4.57
TC12	50,397	1,058	3	5	8	11	19	1568	3.20
TC13	55,581	1,152	4	5	9	14	23	1605	3.78
TC14	50,778	557	3	5	6	7	9	1,050	1.17
TC15	38,313	936	3	4	6	9	13	1668	3.86
TC16	53,662	350	3	3	3	3	3	663	0.69
TC17	47,146	1,031	2	3	6	8	9	1558	3.08
Mean ± *SD*	48,984.12 ± 5,115.26	961.12 ± 271.22	3.06 ± 0.83	4.65 ± 2.18	7.88 ± 3.71	10.88 ± 4.50	15.59 ± 7.31	1517.82 ± 342.56	2.98 ± 1.00

^a^ Observed Chao1 and Shannon indexes were calculated when the sequencing depth was 38,010 reads.

**FIGURE 2:**
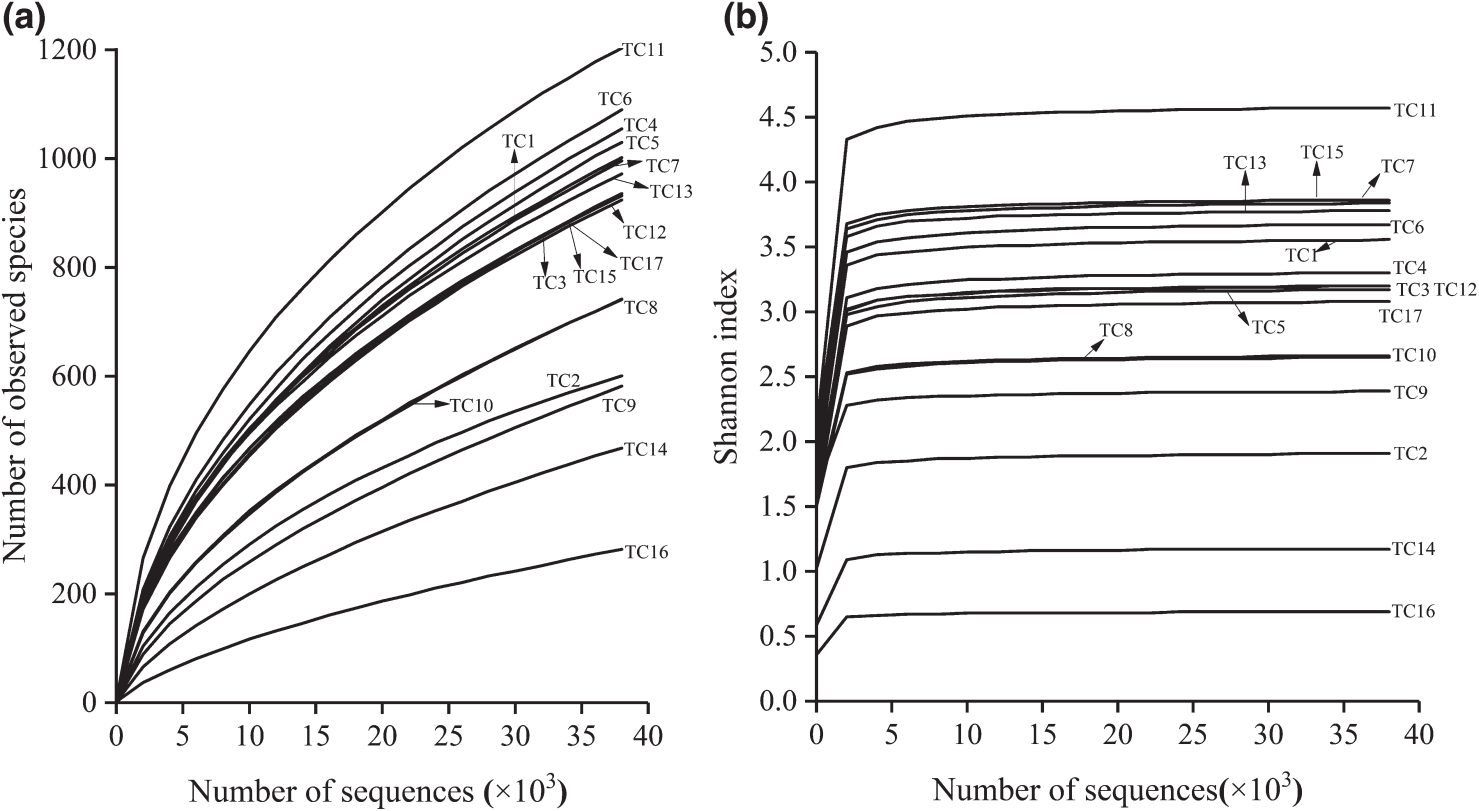
Rarefaction curve (a) and Shannon index curve (b)

By drawing a graph of the number of species found and the Shannon index, the sampling depth and species compositions for each cheese were evaluated, and the species richness and diversity was analyzed (Figure 2). Even when the maximum sequencing depth of a single sample was 56,010, the slope of the dilution curve gradually decreased and tended toward being flat but still displayed an upward trend (Figure 2a). This indicated that as the sequencing depth increases, new bacterial species may appear in the cheese samples. However, when the sequencing depth reached approximately 4,000, the Shannon index curve of all samples became flat and gradually entered a plateau (Figure 2b). This shows that as the sequencing depth increases, new bacterial strains may be discovered, but the diversity of the bacterial communities no longer changes. This indicates that the amount of sequencing data used in this study is reasonable. Increasing the amount of data will make little contribution to the discovery of new OTUs. The existing sequencing depth can better reflect the information of most microorganisms in the sample. Therefore, the average number of 16S rRNA sequences generated per sample in this study (16,827) meets the requirements for subsequent bioinformatic analysis.

### 3.4 Analysis of the relative contents of dominant bacteria in the cheese samples

**FIGURE 3:**
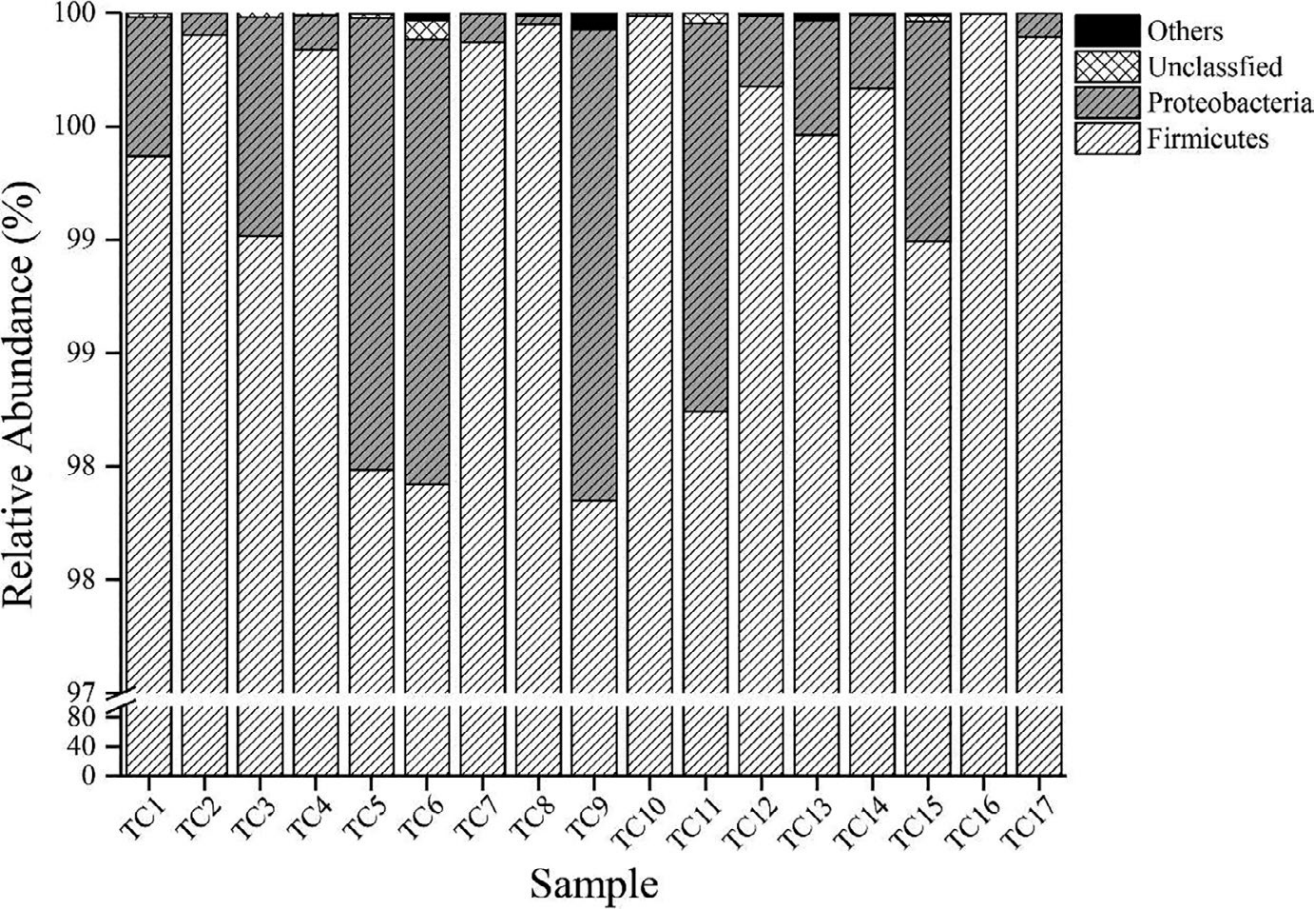
Comparative analysis of relative abundances of bacterial in different phyla between the cheese samples. In this study, the phyla with an average relative abundance of more than 0.1% were defined as dominant, and the rest (except unclassified) were recorded as “others.” Breakpoints were implemented at 97% to improve the presentation of the data

Based on the analysis of the sequence richness and diversity of the cheese samples, a homology comparison of the sequences was carried out after quality control. All sequences were identified within 5 phyla, 12 classes, 19 orders, 23 families, and 32 genera. Dominant bacterial phyla were defined as those with an average relative content of more than 0.1% in the 17 cheeses. The comparative analysis of the relative contents of dominant phyla in the different cheese samples is shown in Figure 3.

According to the Silva database, among the 832,730 optimized sequences, 99.27% of the sequences were identified as Firmicutes and 0.70% were identified as Proteobacteria. The cumulative average relative content of the two was as high as 99.97%, with only 0.01% of the sequences being unidentifiable (Figure 3). It can be concluded that the dominant bacteria in the 17 cheese samples belonged to Firmicutes and Proteobacteria. Firmicutes displayed the highest average relative content in all the samples and was defined as the core bacterial group. In order to further understand the correlation between the distribution of the bacterial groups and the flavor and taste of the cheese, a statistical analysis was conducted for the bacterial genera with an average relative content greater than 0.10%.

**FIGURE 4:**
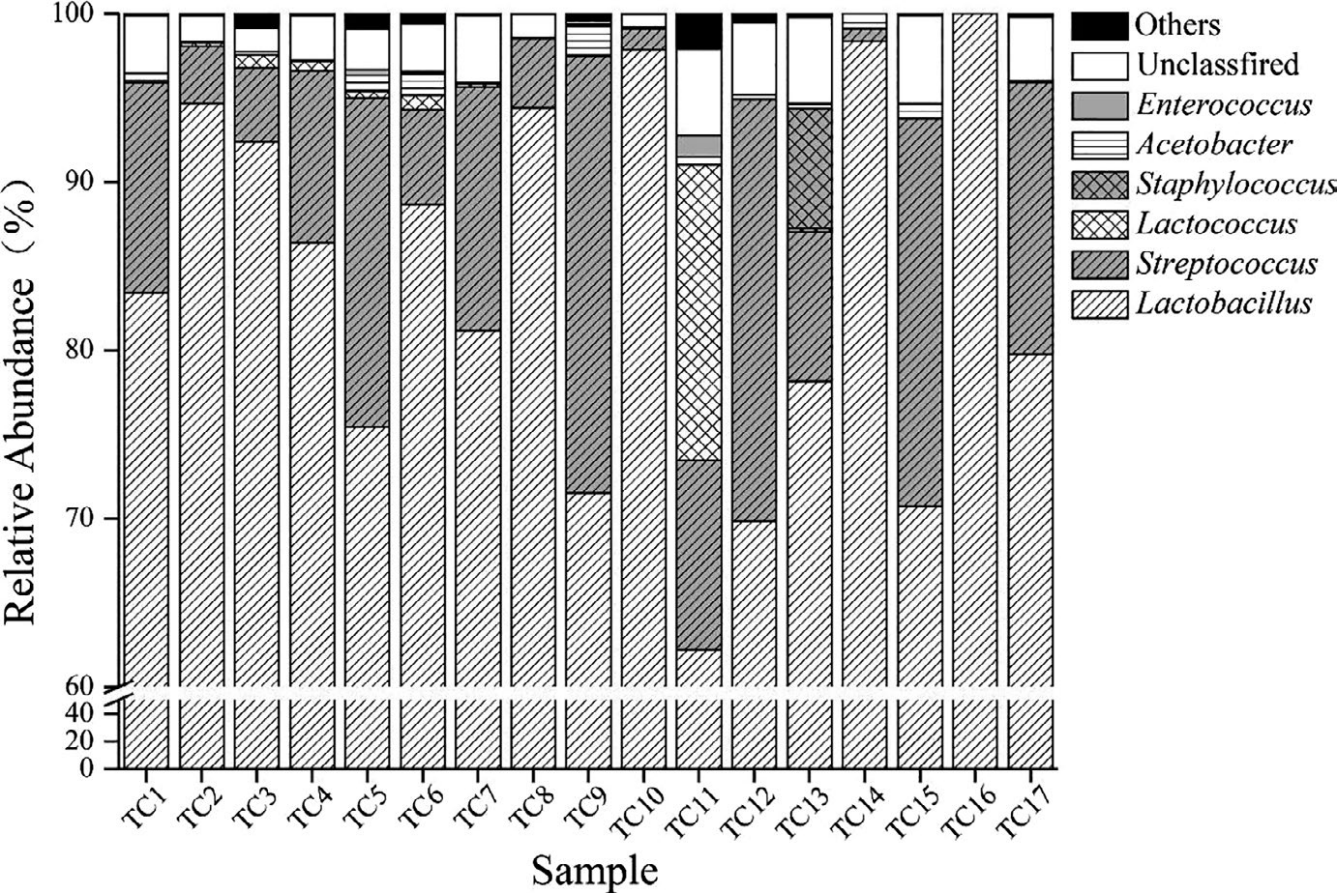
Comparative analysis of relative abundances of bacterial in different genera between the cheese samples. In this study, the genera with an average relative abundance of more than 0.1% were defined as dominant, and the rest were recorded as “others” with the exception of unclassified genera

As shown in Figure 4, the dominant bacterial genera in the traditional Xinjiang cheese samples included *Lactobacillus* (83.81%), *Streptococcus* (10.98%), *Lactococcus* (1.23%), *Staphylococcus* (0.43%), *Acetobacter* (0.42%), and *Enterococcus* (0.12%). *Lactobacillus* and *Streptococcus* were the most dominant bacterial genera, and the cumulative average relative content of the two reached 94.78%. The relative content of unidentifiable sequences in the 17 samples at the genus level was 2.64%. Therefore, most of the bacteria in the 17 samples belonged to *Lactobacillus* and *Streptococcus* of Firmicutes. Researchers used MiSeq technology to analyze the bacterial colonies of cheese in Altay, Beitun, and Emin, Xinjiang, China, and found that 6 of the 10 dominant bacterial genera are consistent with this study, but Ralstonia, Anoxybacillus, Escherichia‐Shigella, and Acinetobacter are four bacterial genera. It was not found in this study. The above‐mentioned common flora is a common species in most traditional handmade cheeses in Xinjiang. However, due to differences in production methods and geographic locations, the structure of the microbial community in the cheese may be different (Wang et al., 2019). As shown in Figure 4, *Lactobacillus* was the only dominant genus in the TC16 sample, which is consistent with the results shown in Table 2. This agreement helps to verify the accuracy of the MiSeq technology in determining the microbial diversity of the samples. The identification of *Staphylococcus* was also important. Within this genus is *Staphylococcus aureus*, which is a common pathogen with clinical manifestations such as skin tissue infection, pneumonia, and osteomyelitis. However, due to the emergence of multi‐drug resistance, it is difficult to treat *Staphylococcus aureus* infections (Lowy, 1998). Therefore, high‐throughput sequencing results can provide information on the safety of traditional fermented foods.

**FIGURE 5:**
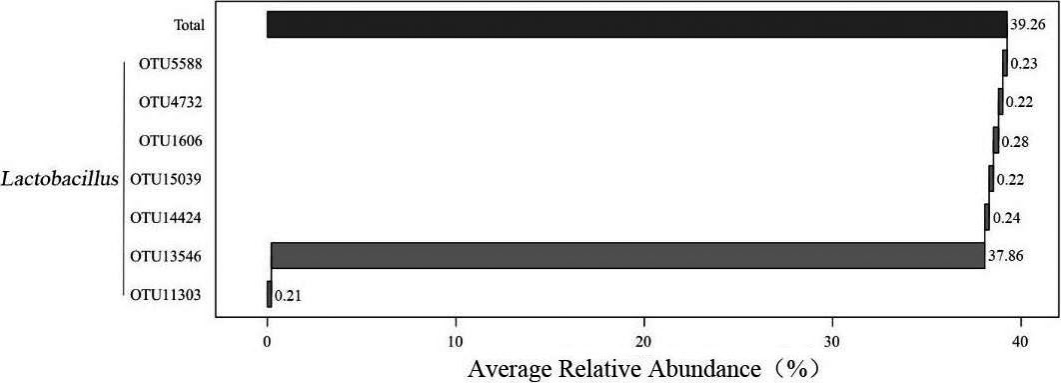
Comparative analysis of the relative contents of the core dominant bacterial OTUs among the 17 cheese samples

On the basis of the data analysis, the total OTU number was further analyzed and seven core OTUs were obtained (Figure 5). The average relative contents of OTU13546, OTU1606, OTU5588, OTU14424, OTU4732, OTU15039, and OTU11303 were 37.86%, 0.28%, 0.23%, 0.24%, 0.22%, 0.22%, and 0.21%, respectively. Among them, OTU13546, which belongs to *Lactobacillus*, was observed in the highest proportions and was the most dominant bacteria found in the cheese samples. This is consistent with the results of the genus‐level analysis and with the results of some previous studies (Zheng, Liu, Li, et al., 2018). It can be concluded that there is a bacterial isolate that was common to all 17 cheese samples, the relative content of which accounted for 38.65% of the total OTU number. The elucidation of this bacterial isolate will be beneficial for further analysis of the relationship between bacterial community and cheese quality.

### 3.5 Analysis of the correlation between cheese quality and bacterial communities

**FIGURE 6:**
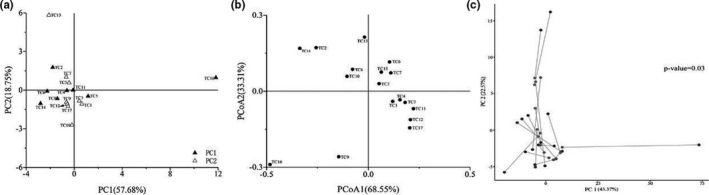
Correlation analysis of cheese quality and bacterial community structure. The score chart of cheese quality factor (a); PCoA based on UniFrac distances between OTUs (b); Procrustes analysis chart of cheese quality and bacterial group (c)

The correlation analysis showed how the cheese samples were distributed in the four quadrants (Figure 6a,b). The samples TC16, TC13, TC2, and TC14 showed significant discrete trends. Meanwhile, TC4, TC1, TC15, TC11, TC12, and TC17 displayed an obvious aggregation tendency and TC5, TC6, TC7, TC8, and TC10 displayed a similar aggregation tendency. These results indicate that there are some differences in the bacterial community structure among the samples. Thus, there may be specific relationships between cheese quality and microbial community structure (Figure 6c). According to Procrustes analysis, cheese quality was significantly correlated with the distribution of bacterial between samples (*p *= .03). According to the analysis of the bacterial diversity in the cheese, different samples were associated with the same dominant bacterial groups. Therefore, it is of great significance to further analyze the correlation between cheese quality and the dominant bacteria present in the cheese.

### 3.6 Analysis of the correlation between specific bacterial groups and cheese quality

**FIGURE 7:**
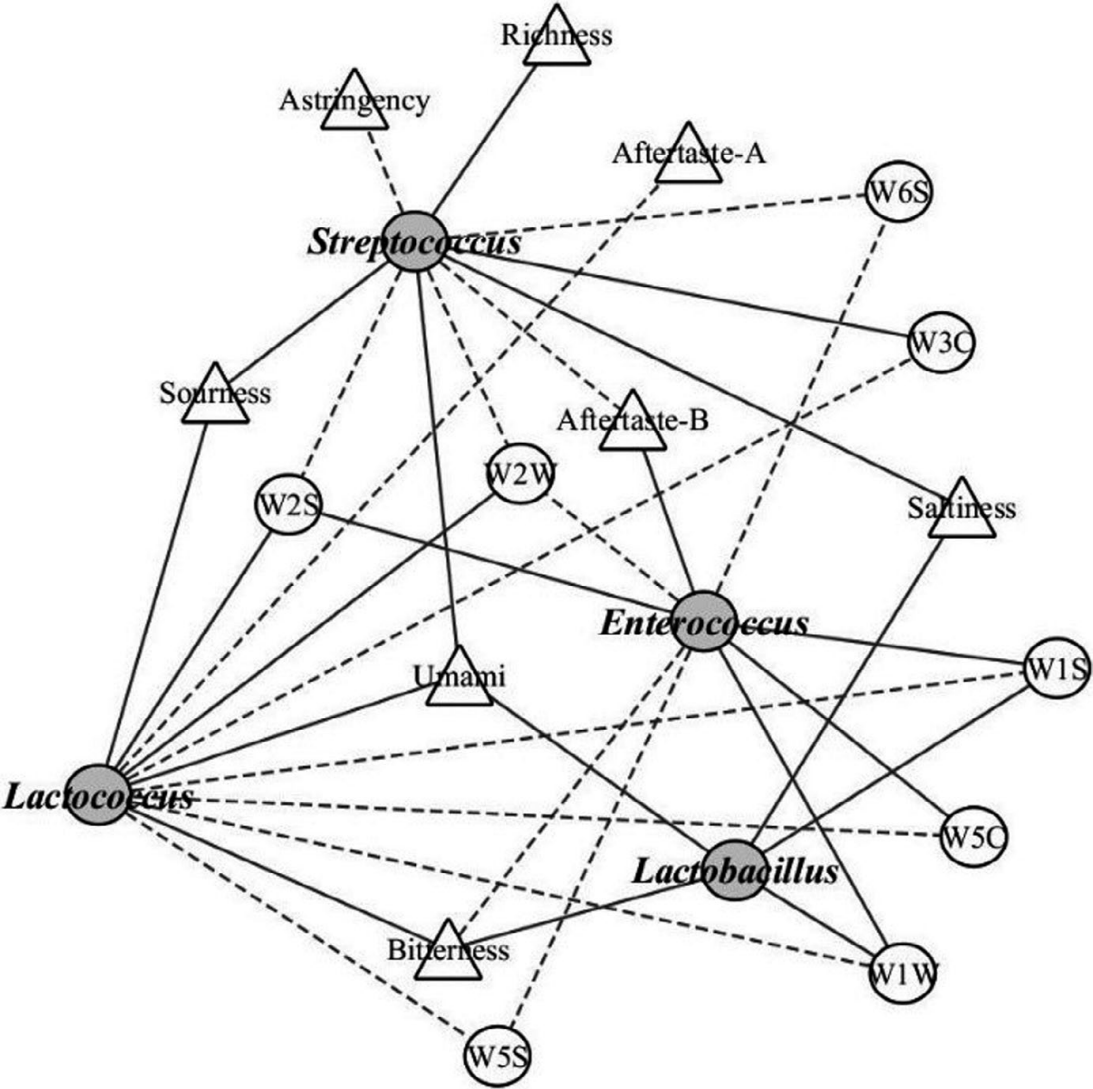
Correlation analysis of dominant bacteria with cheese taste and flavor. Solid circles indicate dominant bacterial genera; the hollow circle indicates the electronic nose sensor; the triangle represents taste index; straight lines indicate positive correlation; and dotted lines indicate negative correlation

The dominant bacteria obtained by high‐throughput sequencing were analyzed for their correlation with taste and flavor. A correlation was deemed significant if *p* <.05 and the absolute value of the linear correlation coefficient R was greater than 0.5. As shown in Figure 7, there was strong correlation between the dominant bacterial genera and the eight taste indexes and eight flavors in the cheese samples. *Lactococcus* displayed the highest positive correlation with sourness, bitterness, and umami (*p* <.05, *R* > .7). Meanwhile, *Lactobacillus* displayed the highest positive correlation with salty taste, umami, and abundance (*p* <.05, *R* > .7). Aftertaste‐B only positively correlated with *Enterococcus* (*p* <.05, *R* > .9) and astringency only correlated with *Streptococcus*, and this was a negative correlation (*p* <.05). W3C and W5C had the strongest correlations with *Streptococcus* and *Enterococcus*, respectively (*p* <.05, *R* > .7). *Enterococcus* displayed the strongest positive correlations with W5S, W1S, and W1W (*p* <.05, *R* > .7). *Lactococcus* displayed the strongest positive correlation with W2S and W2W (*p* <.05, *R* > .9). *Lactobacillus* only displayed a significant positive correlation with W1S and W1W (*p* <.05, *R* > .6). However, the four dominant bacterial genera displayed no significant correlation with W1W or W3S (*p* >.05). Studies have shown that the enzymes produced by *Lactococcus* metabolism participate in amino acid conversion (Gerrit et al., 2010), and promote the formation of cheese flavor. Studies have been conducted to isolate *Lactobacillus* species from cheese; among such species *Lactobacillus plantarum* has been shown to affect the aroma production of cheese during the fermentation process (Diriisa et al., 2019).

## 4 CONCLUSION

In this study, the bacterial diversity of traditional handmade cheese from Tarbagatay, Xinjiang, was analyzed using Illumina MiSeq technology. The taste and flavor of the cheese samples were measured using an electronic tongue and an electronic nose. The taste and aroma of the different traditional cheese samples were found to differ.

In the present study, *Lactobacillus*, *Streptococcus*, and *Lactococcus* accounted for the main proportion of the bacteria, while *Staphylococcus*, *Acetobacter*, and *Enterococcus* accounted for a smaller proportion. OTU13546, which belongs to the genus *Lactobacillus*, was the core dominant bacteria found in the cheese. Meanwhile, the identification of *Staphylococcus*, which accounted for a proportion of 0.43%, was also important. Therefore, the use of high‐throughput sequencing technology provides a basis for investigating both the diversity and the safety of microorganisms in traditional fermented foods.

Based on the results of Procrustes analysis in this study, the cheese quality was found to have a significant correlation with the bacterial flora among the samples. Further positive correlations were found between the dominant bacteria in the cheese and the taste and flavor of the cheese. *Lactococcus* displayed the strongest positive correlations with the sourness, bitterness, umami, W2S, and W2W of the cheese. *Lactobacillus* displayed strong positive correlations with the saltiness, umami taste, abundance, W1S, and W1W in the cheese. Studies have been conducted to isolate *Lactobacillus* species from cheese; among such species *Lactobacillus plantarum* has been shown to affect the aroma production of cheese during the fermentation process (Diriisa, M., et al., 2019). The results show that lactic acid bacteria play a unique role in the fermentation and production of cheese. Overall, the methods and results of this study provide a basis for exploring the diversity of bacteria in cheeses from the Xinjiang Tarbagatay area and lay a foundation for studying the relationship between bacterial communities and cheese quality.

## CONFLICT OF INTEREST

The authors declare that they have no conflicts of interest.

## ETHICAL REVIEW

This study does not involve any human or animal testing.

## INFORMED CONSENT

Written informed consent was obtained from all study participants.
